# Exploring prevalent injuries among tennis players and optimal rehabilitation approaches: A systematic review protocol

**DOI:** 10.1371/journal.pone.0309232

**Published:** 2024-11-07

**Authors:** Eva María Rodríguez-González, María Soledad Amor-Salamanca, Domingo Rosselló, María de Lluc-Bauza, Francisco Hermosilla-Perona, Adrián Martín-Castellanos, Ivan Herrera-Peco

**Affiliations:** 1 Faculty of Health Sciences, Universidad Alfonso X el Sabio, Madrid, Spain; 2 Rafa Nadal Academy, Manacor, Mallorca, Spain; University of Hertfordshire, UNITED KINGDOM OF GREAT BRITAIN AND NORTHERN IRELAND

## Abstract

**Introduction:**

Tennis is a globally popular sport known for its numerous health benefits. However, it also underscores the physical demands and potential injuries associated with high-performance play. This review emphasizes the role of kinetic chains in executing powerful movements and discusses common injuries, particularly in the upper limbs due to the sport’s overhead nature. It highlights the importance of effective rehabilitation methods for swift recovery and long-term performance enhancement in high-performance tennis players.

**Aims:**

The review aims to investigate the relationship between age, sex, and injury prevalence among high-performance tennis players to inform injury prevention strategies.

**Methodology:**

This review protocol will provide a description on effective rehabilitation methods to tennis players, aiding coaches, physiotherapists and physicians. Methodologically, this systematic review will develop following the PRISMA guidelines, focusing on articles published between 2011 and 2024, with eligibility criteria specified. Data collection involved screening titles and abstracts, removing duplicates, and assessing full texts for eligibility. Data extraction will include information on authors, publication year, evidence level, participant demographics, injuries, treatments, etc. The GRADE framework will be used for evidence quality assessment, and NIH criteria were applied for study quality assessment.

**Trial registration:**

**Prospero registration number:** CRD42023453182.

## Introduction

Today, tennis is one of the most practiced sports around the world counting with the participation of 213 national associations affiliated with the International Tennis Federation [1]. There are an estimated 87 million people practicing this sport around the world [2]. The regular practice of tennis promotes a multitude of health benefits, and its dynamic nature improves the cardiovascular fitness, agility and coordination of tennis athletes who practice it [3].

Tennis is an extremely demanding sport that requires repetitive movements for extended periods, leading to significant stress on muscles and joints [4]. In addition, tennis players must move around the court efficiently, running, stopping, and changing direction at any time [5]. The importance of the kinetic chain in tennis, which refers to the interconnected sequence of movements involving various body parts that work together to produce a forceful and coordinated action, plays a critical role in the execution of powerful and precise movements, essential to achieve optimal performance on the court [6].

The optimal coordination of these kinetic chains separates a competent player from an exceptional one [7], from the initial force generation in the lower extremities, through the transfer of energy via the trunk, to the culminating acceleration of the upper extremities. In this sense, for example, the serve represents one of the most important movements in tennis today. The movement requires [8] the efficient transfer of forces through these chains from the lower extremities through the trunk to the lower extremities [9]. The kinetic energy produced helps to generate racquet speed, optimize serve power and improve stroke accuracy, which directly influences the overall game [6]. Thus, the kinetic chain in tennis in tennis can result in both lower and upper extremity injuries [10].

The role of both the upper and lower limbs as well as the core, defined as muscles of the abdominal wall, back extensors, quadratus lumborum, diaphragm, and pelvic floor [11], in playing tennis means that injuries in these areas can appear frequently. It has been described in the literature chronic or overuse injuries appear more frequently in the upper limbs, since tennis is considered an overhead sport [12, 13]. The most frequently described injuries, among others, are elbow’s injuries like lateral epicondylitis (tennis elbow) or medial (golfer’s elbow), shoulder joint injuries, among others. Injuries in the lower limbs are also observed, but more often acute injuries such as sprains or muscle strains at ankle or knee level [9–12, 14, 15]. Regarding the core, it is important to note its role in maintaining body balance and spinal posture [16] which are essential for the proper performance of tennis players [2, 15]. Furthermore, improving core strength significantly enhances athletic performance and reduces injury risk [15, 17], beside functional alteration in the abdominal musculature may increase the risk of shoulder and upper extremity injuries [14].

The link between the kinetic chain and injury risk in tennis is fundamental for understanding and preventing health issues in players. The kinetic chain refers to the sequence of movements and the interaction of body segments to generate and transfer forces during physical activities, like tennis. Efficiency or dysfunction in any of these segments can significantly influence injury risk. Tennis players of all ages use similar movement patterns and kinetic chains, but differences in bone and muscle development between adolescent and adult players affect the types of injuries observed [18]. Adolescents, due to their developmental phases, are more susceptible to certain injuries like tendinopathies, as their bones and muscles are in a growth phase, resulting in increased tension and stress on tendons [19]. Furthermore, studies by Girard et al. (2007) [20] show that fatigue can alter the efficiency of the kinetic chain, increasing injury risk. The biomechanics of the serve, a critical movement in tennis, is closely linked to the kinetic chain and can be a significant source of injury if not executed correctly [21].

This situation necessitates a comprehensive understanding of optimal recovery processes to ensure the tennis player quickly returns to pre-injury form. This includes effective injury rehabilitation and a gradual reintroduction to physical exercise [20] to rebuild muscle coordination and enhance overall performance [22–24]. These types of approaches and the proper development of coordination between approaches facilitates a swift return to the court but also contributes to prevent injuries and protect players’ health, the long-term success and well-being of the athlete [25].

### Aims

This review will highlight the importance of examining the possible relationship between age and sex and the prevalence of injuries in high-performance tennis players. Understanding these relationships is crucial because age and sex are fundamental factors that can influence injury risk and recovery processes.

This knowledge will aid in guiding clinical decision-making and designing injury prevention programs tailored to the specific needs of tennis players.

The review aims to support both primary and secondary prevention efforts, ultimately enhancing the health and performance of high-performance tennis athletes.

## Materials and methods

A systematic review will be performed based on the criteria of PRISMA statement.

### Eligibility criteria

The following criteria will be defined to retrieve eligible studies for this review. Inclusion criteria will be defined as: i) articles written in English, ii) articles published in a peer-reviewed journal, iii) studies with a cross-sectional design iv) articles with participants who are adolescents (13 to 18 years of age) and adults as professional players. v) articles published between January 1, 2011, and September 1, 2024.

Exclusion criteria will be defined as i) non-research articles (editorials, commentaries, preprints, abstracts, proceedings, book reviews, etc.), ii) reviews or systematic reviews, iii) articles based on other sports, like football, basketball, rugby, etc. iii) articles focusing on other racquet sports, like beach tennis, table tennis, paddle, etc., iv) Articles with participants 6 to 12 years of age, v) articles involving adolescent and adult participants who play tennis at an amateur level.

### Search methodology

To identify studies of interest, a bibliographic search will be conducted covering a wide range of published health-related research from the following databases: Medline, Web of Science, Cochrane Library and Scopus. The time frame of the study will include articles published from January 1, 2011, and September 1, 2023.

The search terms used in the different databases will be focused on obtaining the appropriate result to answer the objectives set out in this study. These will be as follows: terms used for risk factors of tennis injuries, will include “prevention” OR “incidence” OR “risk factors”, Furthermore, search terms related to injuries like “injury” OR “wounds” OR “injuries”, and finally terms linked to tennis, like “tennis” OR “tennis players”

### Data collection

After retrieving the studies from the databases, duplicate reports will be removed, and the titles and abstracts of the remaining articles will be screened to exclude studies that did not meet the eligibility criteria. To avoid error and bias, three independent researchers will conduct the review process to identify articles that will meet the inclusion criteria, using the Zotero bibliographic reference manager, which will allow for the detection and elimination of duplicate articles [26]. Titles and abstracts will be then analyzed to exclude irrelevant articles. Finally, the full texts will be evaluated using PRISMA criteria, to determine whether the articles will meet the eligibility criteria. The quality assessment according to the PRISMA criteria among the researchers will be evaluated by analyzing the reliability with Cohen’s kappa coefficient. GRADE Framework (Grading of Recommendations, Assessment, Development, and Evaluation) system will consider various types of studies in assessing the quality of evidence [27]. This comprehensive approach will ensure clinical recommendations are grounded in the best available evidence, bolstering informed and evidence-based decision-making in the realm of healthcare.

During this selection phase, any disagreements among the investigators will be resolved by discussion and consultation with a reviewer who will not be actively involved in the study selection.

### Data extraction

The following data will be extracted from the selected articles; I) authors, ii) year of publication, iii) Level of evidence based on GRADE framework, iv) population/participants, v) country, vi) injuries, vii) location of injuries, viii) severity of injuries, ix) treatments to injuries.

### Quality assessment

The quality assessment for each study will be assessed using the tool of the National Heart, Lung, and Blood Institute (NIH) (https://www.nhlbi.nih.gov/health-topics/study-quality-assessment-tools) for cross-sectional studies that include 14 criteria [28]. Three independent researchers will assess each article. We will rate the studies as ‘Good’ if the count was ≥11, ‘Fair’ if the count was between 5 and 10, and ‘Poor’ if the count is ≤4.

## Registration

This Review protocol was registered previously in PROSPERO (ID. CRD42023453182).

## Supporting information

S1 File PRISMA for systematic review protocols (PRISMA-P). (DOCX)

S2 File Search strategy example. (DOCX)

S1 Fig PRISMA Flow diagram. (DOCX)
