# Correction: Exploring prevalent injuries among tennis players and optimal rehabilitation approaches: A systematic review protocol

**DOI:** 10.1371/journal.pone.0327328

**Published:** 2025-06-30

**Authors:** Eva María Rodríguez-González, María Soledad Amor-Salamanca, Domingo Rosselló, María de Lluc-Bauza, Francisco Hermosilla-Perona, Adrián Martín-Castellanos, Ivan Herrera-Peco

An additional affiliation is missing for the fifth author. Francisco Hermosilla-Perona is also affiliated with the Facultad de Ciencias de la Vida y la Naturaleza, Universidad Nebrija, Madrid, Spain.
