# Supplementary material for: Exploring prevalent injuries among tennis players and optimal rehabilitation approaches: A systematic review protocol
Source: PLoS One. 2024 Nov 7;19(11):e0309232. doi: 10.1371/journal.pone.0309232 (PMC11542848; doi:10.1371/journal.pone.0309232)
Supplement: S2 File — (DOCX) [file pone.0309232.s002.docx]

SEARCH STRATEGY

An example of the search syntax used in SCOPUS search engine was as follows:

(prevention OR prevent* OR incidence OR aetiology OR risk factors) AND (Injury OR Injur* OR wounds) AND (adolescents OR young adults OR adults) AND (tennis players OR tennis) NOT (tennis table OR beach tennis)
